# Comparison of active tuberculosis case finding strategies for immigrants in South Korea: Epidemiology and cost-effectiveness analysis

**DOI:** 10.1371/journal.pone.0283414

**Published:** 2023-04-20

**Authors:** Sangwook Park, Chaegyu Sung, Hangseok Choi, Yeo Wool Lee, Yedham Kang, Hee-Jin Kim, Hae-Young Kim, In-Hwan Oh, Seung Heon Lee

**Affiliations:** 1 Korea University College of Medicine, Seoul, South Korea; 2 Medical Science Research Center, Korea University Ansan Hospital, Ansan, South Korea; 3 Department of Preventive Medicine, Korea University College of Medicine, Seoul, South Korea; 4 Division of Pulmonary, Sleep and Critical Care Medicine, Department of Internal Medicine, Korea University Ansan Hospital, Ansan, South Korea; 5 Central Education Institution, KoreanNational Tuberculosis Association, Seoul, South Korea; 6 Department of Population Health, New York University Grossman School of Medicine, New York, New York, United States of America; 7 Department of Preventive Medicine, Kyung Hee University College of Medicine, Seoul, South Korea; Tribhuvan University, NEPAL

## Abstract

**Background:**

Tuberculosis (TB) is one of the serious infectious diseases in South Korea, with 49 new cases per 100,000 people and 629 multi-drug resistant (MDR) cases reported in 2020. TB is increasing among immigrants in S. Korea, and various TB case finding strategies are being performed for screening. We compared active case finding (ACF) with passive case finding (semi-PCF) across epidemiological characteristics and investigated a cost-effective strategy for screening immigrants for TB.

**Methods:**

ACF driven by non-governmental organizations and semi-PCF as part of the government’s visa renewal process using CXR with additional acid-fast bacilli (AFB) smear and cultures were performed. Epidemiological parameters were compared between the two TB screening projects, and costs were collected. Cost-effectiveness was evaluated using a decision analysis model from the health system perspective. The primary outcome was incremental cost-effectiveness ratio (ICER) per averted TB case. Additional probabilistic sensitivity analysis was conducted.

**Results:**

ACF (2.02%) showed a higher TB prevalence rate than semi-PCF (0.67%) on CXR. For subjects older than 60 years, the suspected TB rate on CXR was significantly higher in ACF (36.6%) than in semi-PCF (12.2%) (P<0.01). TB incidence among the family visa type was significantly higher in ACF (1.96%) than in semi-PCF (0.88%) (P < 0.0012). Costs for ACF ($666.92) were $20.784 higher than for semi-PCF ($646.13), but TB progression decreased by 0.02, resulting in an ICER of $948.18 per averted TB case. In sensitivity analysis, the indirect costs of ACF and semi-PCF had the highest impact on ICER.

**Conclusion:**

ACF found more TB cases than semi-PCF through CXR screening, and suspect cases with old age and family visa type were more common in ACF than in semi-PCF. ACF is cost-effective as a TB screening strategy for immigrants.

## Introduction

Tuberculosis (TB) is the leading cause of mortality among infectious diseases, accounting for 1.3 million deaths worldwide in 2020 [1]. South Korea (S. Korea) had a TB incidence rate of 49 cases per 100,000 in 2020 and the third-highest TB mortality rate among all Organization for Economic Cooperation and Development (OECD) countries at 3.8 cases per 100,000 in 2020 [2]. Among immigrants, annual TB incidence increased and peaked at a total of 2,569 cases in 2016 and then started declining after the Korean government began to require all foreigners from high TB burden countries to receive TB screening for renewal or upgrade of their visa in 2016. TB screening and management in immigrants are important in the economy of S. Korea. The economic burden for drug-susceptible-TB (DS-TB) and multidrug-resistant-TB (MDR-TB) in 2017 was $116.62 million and $408.14 million, respectively, and MDR-TB cases from immigrants accounted for nearly 20–30% of total MDR-TB cases in S. Korea in recent annual reports [3].

Visas for overseas Koreans are granted to Korean-Chinese and Korean-Russians whose ancestors were mobilized by force to engage in the Chinese-Japanese War and Russian-Japanese War and who are becoming naturalized as Korean citizens according to the special policy for overseas Koreans [4]. In addition, many migration workers with an employment visa from East Asia, Central Asia, and China are working in S. Korea to become naturalized as citizens. However, as most such immigrants are from high-TB burden countries, the Korean government scaled up the TB control policy to include intensive TB screening for immigrants [5, 6]. This is in addition to the many non-governmental organization (NGO)-based medical screening services in immigrant communities that offer CXR and other serologic diagnostic tools. However, there have been no standardized reports or rules about TB diagnosis and treatment algorithms for immigrants until now.

The TB case finding strategy is one of the control policies that reduces the TB incidence in high-burden countries. TB case finding methods can be divided into ACF and PCF [7]. ACF is defined as a provider-initiated strategy to identify and treat TB patients who would not otherwise receive prompt medical service. ACF screens mass populations for active TB usually with CXR, which is very resource intensive. On the other hand, PCF identifies active TB following the voluntary presentation of a symptomatic patient to a health care provider. However, there are several controversies about the appropriateness (TB detection rate, TB detection timing, treatment outcomes, and economic and social consequences) of ACF and PCF depending on the epidemiology, the health system context, and resource availability [8].

Regarding the screening tools, CXR has been a primary tool for detection of TB since it has high sensitivity (87% - 98%) despite its low specificity (46% - 89%) [8], depending on interpretation, and it provides rapid results in addition to easy mobility. However, for CXR, there have been concerns about costs, workload, and availability of infrastructure and qualified staff. But, owing to digitalized portable equipment [9] and its own confirmative diagnostic values, CXR can be beneficial, weighing the tradeoff between concerns and benefits to the vulnerable groups. Therefore, the strategy of effective case finding with CXR in addition to other additive diagnostics must be suggested based on epidemiologic outcome data and cost-effectiveness.

In this study, we aimed to compare and reassess active and passive TB case finding for immigrants from the perspective of public health and to determine a cost-effective TB screening strategy for immigrants in South Korea.

## Materials and methods

### TB screening strategies from previous projects

**Fig 1:**
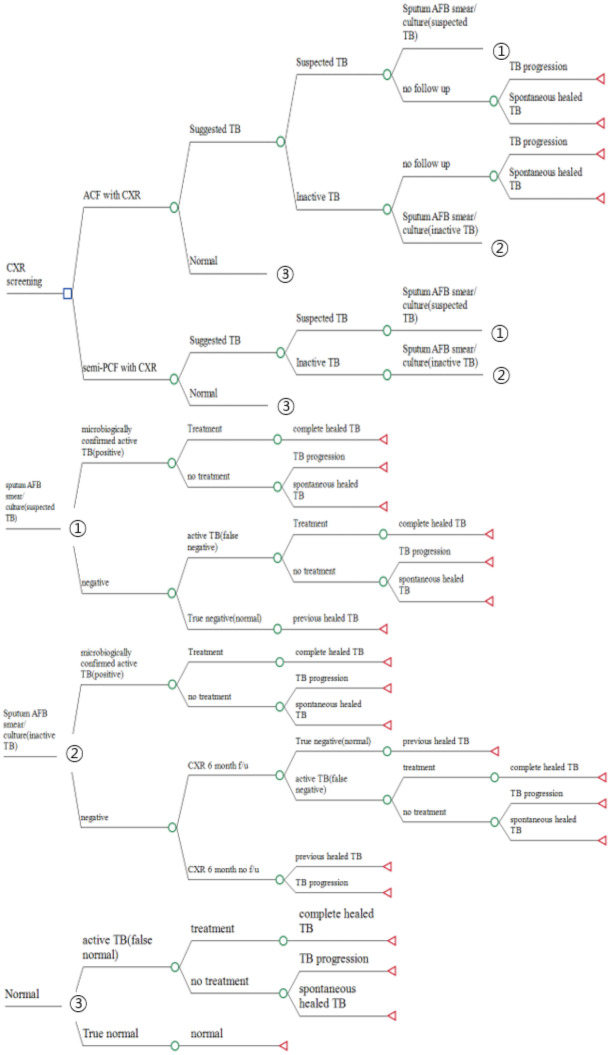
TreeAge model outline for ACF and semi-PCF. Each branch represents one possible instance. The comment below the node indicates the possibility of each node. The total cost of the branch is written next to the terminal node. CXR = chest X-ray, ACF = active case finding, semi-PCF = passive case finding, F/U = follow-up, TB = tuberculosis, SC = sputum culture test, AFB = acid-fast bacillus.

Many NGOs began conducting ACF in 2014 as a TB screening project for immigrant workers and multicultural families based on the 2013 government policy of early TB detection and infection prevention in vulnerable groups [10]. In contrast, we defined the administrative screening for foreigners seeking visa renewal in 2018 and 2019 as semi-PCF, because this screening was obligatory even though the participants had no related symptoms [11]. These strategies differ in its method of TB case finding, but both of them are used as similar cascades after CXR screening. CXR was initially used to classify the subjects into ‘normal’, ‘suspected TB’, and ‘Inactive TB’. And then, ‘Suspected TB’ cases are further inspected with sputum AFB smear/ culture to confirm active TB. As some of cases may have false negative results, all corresponding cases are classified into each responding decision tree (Fig 1). Both projects were carried out by the Korean Centers for Disease Control and the Korean National Tuberculosis Association (KNTA). We reviewed the epidemiologic reports and reanalyzed the data from the original reports for comparison. In addition, for cost-effectiveness analysis, the probability of epidemiologic variables were input as well as other parameters including cost described below.

### Epidemiological parameters and cost measurement

Epidemiological parameters relevant for our models were based on the above-mentioned TB screening projects and literature review. For TB screening, diagnosis, and treatment, we estimated the costs using both top-down and bottom-up approaches. Cost-related data and statistics were collected through the operation’s financial reports. Common programmatic costs (indirect and overhead costs) and human resources data were first calculated as total costs and were apportioned into each category. We estimated the time, resources, and unit prices required to conduct each screening and follow-up visit, diagnosis, and treatment based on national TB management guidelines. The unit cost for TB diagnosis with treatment per patient was recalculated from the direct costs of the related diagnostic tools and medications and from the patient service utilization data of the National Health Insurance Review and Assessment Service [12]. Capital costs (e.g., mobile X-ray vehicle) were annuitized based on a 3% discount rate and expected life-years of the equipment [13]. The cost of active TB treatment includes the cost of testing and drugs over two months of the intensive phase, including isoniazid (H), rifampicin (R), ethambutol (E), and pyrazinamide (Z) (2HREZ), and over 4 months of the maintenance phase, including isoniazid, rifampicin, and ethambutol (4HRE). Costs were collected as Korean Won (￦) and were converted into 2021 US dollars ($) using the standard inflation adjustment method, at the rate of 1133.5 Won per US$1, based on the average UN exchange reported for 2021, April 7 [14]. We did not discount our cost and effectiveness estimates, as the assumed time frame of our study was only 2 years.

### Decision tree and scenarios

A simplified outline of the decision tree represented through the TreeAge model is shown in Fig 1. Each branch represents possible instances from the previous stage, resulting in different responses and TB progression, following the corresponding possibility and cost of the branch.

The decision tree was divided into two scenarios, ACF and semi-PCF. The two programs used initial CXR screening but with different methods of recruiting participants. Each participant was first tested with CXR and then classified as ‘suggested TB’ or ‘normal’ according to the CXR result. Suggested TB included suspected TB, inactive TB, and equivocal with observation branches. The suggested TB group was then tested with a sputum AFB smear culture test to confirm TB biologically, proceeding to treatment if the result was positive. If the patient had inactive TB and was negative on the sputum AFB smear, the patient was followed in 6-month intervals. Even if a patient was suspected with TB on CXR but was negative on SC, the patient could get treatment, as the SC result could be a false negative. If the SC result was a true negative, the patient was regarded as having previously healed from TB; therefore, treatment was not required in this scenario. For every instance where a patient with TB failed to get proper treatment due to refusal or nondetection, the patient spontaneously healed or experienced TB progression. These processes were applied in the same way for both ACF and semi-PCF but with different probabilities.

We assumed that 1) all individuals with suspected TB would undergo SC in semi-PCF, 2) five percent of inactive TB subjects with negative SC in both ACF and semi-PCF would be followed up with CXR, and 3) 99% of SC-positive individuals would receive treatment in both ACF and semi-PCF.

### Statistical analysis and cost-effectiveness analysis

We investigated the differences between the two TB case finding strategies using the t-test and chi square test. P value < 0.05 was considered statistically significant. The cost-effectiveness analysis was performed with the decision tree model using 2020 TreeAge software (Williamstown, MA, USA). Relevant costs for TB screening, diagnosis, and treatment were allocated to each step, and total costs and total TB cases averted were estimated for each outcome. Our main effectiveness measure was total TB cases averted, which was estimated based on the difference between the estimated total number of TB cases without optimal case detection with treatment and the total number of TB cases occurring with optimal case detection with treatment. The cost-effectiveness analysis estimated health outcomes and increment of costs spent on individual participants in the ACF or semi-PCF programs. Differences in cost and health outcomes were calculated into the ICER to determine the cost-effectiveness for the ACF and semi-PCF programs.

### Ethical approval

Consent for this study was waived as it was based on a retrospective reanalysis of data from a previous study. This study was approved by the Institutional Review Board (IRB) of Korea University Ansan Hospital (IRB number: 2022AS0066).

## Results

### Baseline characteristics in TB case finding

Totals of 7,274 and 10,961 immigrants received TB screening through ACF and semi-PCF, respectively. In ACF, 145 cases (2.02%) with suspected TB were reported, and 11 cases (0.15%) were diagnosed with active TB (Table 1).

**Table 1:** Proportion of suspected TB by region of origin in ACF with CXR and semi-PCF with CXR.

	Active Case Finding [10]		Semi-Passive Case Finding [11]		P value
	Suspected TB	Total	Suspected TB	Total	
Gender Female	74 (1.8%)	4,130 (56.8%)	29 (0.64%)	4,551 (41.5%)	<0.01
Male	71 (2.3%)	3,144 (43.2%)	45 (0.70%)	6,410 (58.5%)	<0.01
Total	145 (2.0%)	7,274 (100%)	74 (0.68%)	10,961 (100%)	<0.01
Age (mean)	55.6 ± 11.2		42.8 ± 14.3		<0.01
Among family visa type	57 (1.96%)	2,907	24 (0.88%)	2,701	0.0012
region of origin					
Chinese	81 (2.9%)	2,781 (38.2%)	50 (0.8%)	6,524 (59.5%)	<0.01
Central Asian	3 (1.1%)	276 (3.8%	12 (0.6%)	2,022 (18.4%)	.340
Southeast Asian	53 (1.4%)	3,679 (50.6%)	11 (0.5%)	2,186 (19.9%)	<0.01
Others	8 (1.5%)	538 (7.4%)	1 (0.4%)	229 (2.1%)	.214
Total	145 (2.0%)	*7,274 (100%)	74 (0.68%)	*10,961 (100%)	<0.01

TB = tuberculosis; CXR = chest X-ray

*Active tuberculosis cases were confirmed in 11 of 7,274 (0.15%) subjects in active case finding strategy, and in 18 of 10,961 (0.16%) subjects in semi-active case finding strategy, respectively.

In semi-PCF, 74 cases (0.67%) with suspected TB and 12 active TB cases (0.10%) were confirmed. The primary region of origin for examinees in ACF was Southeast Asian, while semi-PCF examinees were mostly Chinese (P < 0.001).

ACF has shown a higher suspected TB rate in older adults, while semi-PCF tended to show a higher suspected TB rate in younger adults (Table 2).

**Table 2:** Proportion of suspected TB by age group in ACF with CXR and semi-PCF with CXR.

Age (years)	Active Case Finding [10]		Semi-Passive Case Finding [11]		P value
	Suspected TB	Total	Suspected TB	Total	
~19	0 (0.0%)	101 (1.4%)	2 (2.7%)	22 (2.0%)	<0.01
20–29	17 (11.7%)	1,940 (26.7%)	15 (20.3%)	2,377 (21.7%)	.376
30–39	21 (14.5%)	1,962 (27.0%)	21 (28.4%)	2,840 (25.9%)	.226
40–49	24 (16.6%)	1,250 (17.2%)	10 (13.5%)	2,164 (19.7%)	<0.01
50–59	30 (20.7%)	1,056 (14.5%)	17 (23.0%)	2,592 (23.6%)	<0.01
60~	53 (36.6%)	950 (13.0%)	9 (12.2%)	765 (7.0%)	<0.01
N/A	-	15 (0.2%)	-	-	NA
Total	145 (2.0%)	*7,274 (100%)	74 (0.68%)	*10,961 (100%)	

TB = tuberculosis; CXR = chest X-ray

*Active tuberculosis cases were confirmed in 11 of 7,274 (0.15%) subjects in active case finding strategy, and in 18 of 10,961 (0.16%) subjects in semi-active case finding strategy, respectively.

For individuals older than 60 years, the suspected TB rate on CXR was significantly higher in ACF (36.6%) than in semi-PCF (12.2%) (P < 0.01).

### Probability

Probability data for the analysis were collected from the above-mentioned TB screening projects, and the other basal values were collected from literature reviews (Table 3).

**Table 3:** Base-case value, range, and description of variables used in cost-effectiveness analyses for ACF with CXR and semi-PCF with CXR.

Description	Base-case value	Range	Source
False normal in CXR	0.308	0.262–0.354	[15]
Sputum culture false negative	0.427	0.418–0.436	[15]
Inactive TB in suspected TB	0.883	0.750–0.927	[11]
ACF			
CXR normal in ACF	0.978	0.831–0.997	[11]
ACF inactive TB, underwent SC test	0.16	0.136–0.184	[16]
ACF inactive TB, SC negative, CXR F/U	0.05	0.042–0.058	*
ACF inactive TB, SC negative, CXR no F/U, true inactive TB	0.383	0.325–0.440	[17]
ACF inactive TB, SC positive TB	0.004	0.003–0.005	[11]
ACF suspected TB, underwent SC test	0.676	0.575–0.777	[18]
ACF suspected TB, SC positive TB	0.0102	0.0102–0.03125	[18]
ACF suspected TB, no SC test, TB progression	0.16	0.136–0.184	[16]
semi-PCF			
CXR normal in semi-PCF	0.942	0.801–0.990	[10]
semi-PCF inactive TB, SC positive TB	0.004	0.003–0.005	[11]
semi-PCF inactive TB, SC negative, CXR F/U	0.05	0.042–0.058	*
semi-PCF inactive TB, SC negative, CXR no F/U, true inactive TB	0.383	0.325–0.440	[17]
semi-PCF suspected TB, SC positive TB	0.233	0.198–0.267	[19]
SC active TB, yes treatment	0.99	0.842–0.999	*
SC active TB but no treatment, spontaneously healed	0.295	0.25–0.33	[20, 21]

* Assumed in this study

TB = tuberculosis; CXR = chest X-ray; ACF = active case finding; semi-PCF = passive case finding; SC = sputum culture test; F/U = follow-up

Among the 7,274 cases for ACF and 10,961 cases for semi-PCF, 97.8% and 94.2% were normal on CXR. Among the ‘suggested TB’ group after excluding other abnormalities, 88.3% were inactive TB, and 11.7% were suspected TB. The false normal rate of CXR was 30.8%, and 0.4% of the inactive TB was positive on SC.

### Cost

The cost for outpatient clinics included the CXR and sputum AFB smear culture, the peripheral blood smear examination on the first visit and each month during the 6-month follow-up, molecular DST (drug sensitivity test) for Isoniazid(INH) and Rifampicin(RFP), and the phenotypic DST (Table 4).

**Table 4:** Base-case value per patient, range, and description of costs in cost-effectiveness analyses for ACF with CXR and semi-PCF with CXR.

Description	Base-case value ($)	Range ($)	Source
Cost of initial CXR	5.826	5.234–6.930	[16]
Cost of CXR at 6-month F/U	5.826	5.234–6.930	
*Indirect cost of ACF	33.98	28.89–39.08	[10]
*Indirect cost of semi-PCF	26.12	22.20–30.40	[11]
Cost of sputum culture	24.62	20.93–28.64	[22]
**Cost of follow-up with treatment	2,065	1,756–2,376	[22]
Cost for outpatient clinics	330.4		[22]
Cost for active TB treatment	1,734		[22]

CXR = chest X-ray; ACF = active case finding; semi-PCF = passive case finding; F/U = follow-up

*Indirect cost of ACF and semi-PCF includes cost of labor, office rental, and vehicle rental

**Includes cost for screening (CXR, SC, AFB smear) and treatment (2-month intensive phase, 4-month maintenance phase)

The per-patient costs of the digital CXR and sputum culture test were $5.826 (range: $5.234-$6.930) and $24.62 (range: 20.93–28.64), respectively. The total cost for one active TB patient was $2,065 (range: 1,756–2,376), which is the sum of the outpatient clinic expense of $330.4 and the actual medication cost of $1,734. The indirect costs of ACF and semi-PCF were $33.98 (range: 28.89–39.08) and $26.12 (range: 22.20–30.40), respectively.

### Cost-effectiveness analysis

The total cost of ACF with CXR per patient was $666.92, and the cost of semi-PCF with CXR per patient was $646.13. The ICER result of the model was $948.18 per person (Table 5).

**Table 5:** Cost-effectiveness rankings report of the model.

Category	Strategy	Cost per individual ($)	Incremental Cost ($)	Active TB progression ratio	Decreased active TB progression ratio	ICER per active TB case averted
Undominated	Semi-PCF with CXR	646.13		0.03		
Abs.dominated	ACF with CXR	666.92	20.784	0.01	0.02	948.18

TB = tuberculosis; ICER = incremental cost-effectiveness ratio

The cost of ACF has increased approximately $20.784 compared to that of semi-PCF, while the possibility of progression has decreased from 0.03 to 0.01. This implies that ACF can lower TB progression by 0.02 at the expense of $20.784. The estimated ICER was $948.18/averted TB case, showing that it can prevent a single additional TB case from progressing by spending $948.18 with ACF instead of semi-PCF. Compared with the average income (Gross Domestic Product(GDP) per capita) of Korea, which is estimated at $31,637 (2020, KOSIS), this is a reasonable cost to prevent TB.

### Sensitivity analysis

**Fig 2:**
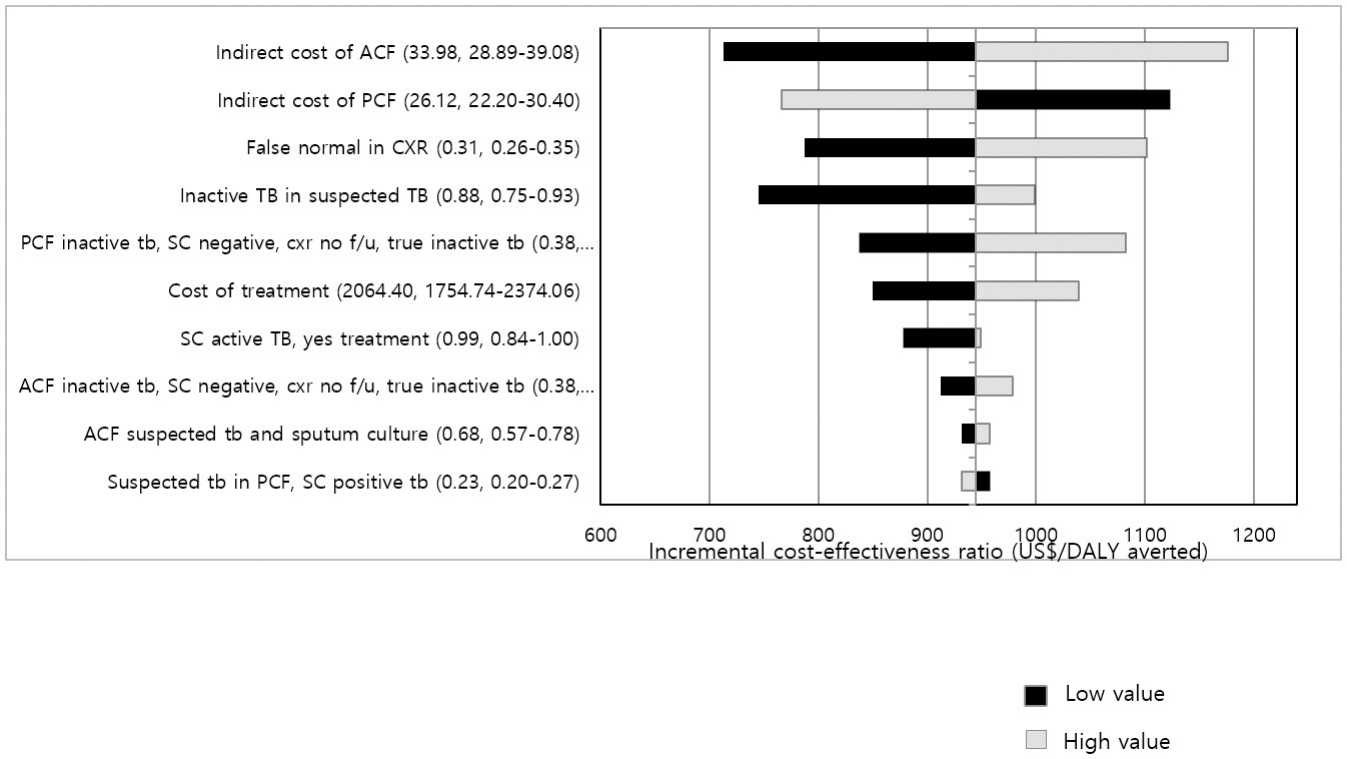
Impact of parameters used in this model on the cost-effectiveness of ACF with CXR and semi-PCF with CXR. The bars represent the incremental cost-effectiveness ratio at the lower (black bar) and higher (gray bar) values for each variable. TB = tuberculosis, CXR = chest X-ray, ACF = active case finding, semi-PCF = passive case finding, SC = sputum culture test.

Analysis on the impact of each key parameter on cost-effectiveness is shown in Fig 2. The parameters are listed in the order of influence against cost-effectiveness. A low value (black color) indicates that cost-effectiveness has a positive correlation with parameter value, whereas a high value (gray color) indicates a negative correlation. As shown in the graph, the indirect costs of ACF and semi-PCF had the greatest impact, followed by other parameters including false normal rate of CXR and proportion of previously healed TB cases (inactive TB in suspected TB). Although each parameter can cause fluctuation in the ICER, its value stays far below the GDP per capita. In probabilistic sensitivity analysis with 1,000 Monte Carlo simulations, at a Willingness-to-pay (WTP) threshold of $1,500, ACF was cost-effective compared with semi-PCF in 100% of instances (S1 Fig).

## Discussion

Based on previous CXR TB screening data of immigrants, ACF showed a higher TB prevalence rate on CXR and a higher suspected TB rate in the older generation compared with semi-PCF. We found that the ACF program for immigrants was highly cost-effective compared to the semi-PCF program. ACF could lower TB progression by 0.02 at the expense of $20.784, resulting in an ICER of $948.18. However, much effort is needed to ensure ACF TB screening for the younger generation who might have TB with higher infectivity.

There were several differences between the ACF and semi-PCF programs, such as the participants’ average age, region of origin, visa type, and CXR-positive ratio. TB prevalence was significantly higher in ACF than in semi-PCF for Chinese (2.9% vs. 0.8%) and Southeast Asian people (1.4% vs. 0.5%). Regarding the age distribution, ACF has shown a higher suspected TB rate in the older population (older than 60 years). A plausible reason for this trend is that most subjects in ACF had immigrated many years ago to South Korea [23]. On the other hand, semi-PCF involved younger immigrants wishing to renew their working visa with TB-free status via CXR screening [23]. However, in ACF, a more aggressive case finding strategy for the younger working generation should be considered to identify hidden infectious TB patients.

As presented in Fig 2 and based on the base-case values from Table 3, diagnostic accuracy with CXR, such as false normal and true normal rates including previous healed TB, is an important element in CXR mass screening. According to the result, 5% of participants had inactive TB with a negative result on SC. This can cause unnecessary treatment since participants with previously healed TB appear as inactive TB with a negative SC result. Therefore, the prevalence of previously healed TB might have a greater impact on TB screening as time passes to differentiate real, active TB cases from inactive TB cases to avoid unnecessary over-treatment [24, 25]. In order to properly deal with such patients in the screening process, collection and utilization of previous medical history are important. In addition, verification by a second chest radiologist is a good option for the national TB screening project to reduce inter-observer variation [24, 25]. As the immigrant population is highly vulnerable, national efforts on securing and managing quality control for screening methods are required.

The cost-effectiveness analysis suggests that the ACF program for immigrant TB screening in S. Korea is highly cost-effective. The ICER of the ACF program ($948.18) is well below the GDP per capita of S. Korea ($31,637) or the average income of immigrant workers ($25,064) in S. Korea (TASIS, 2020) [26]. However, while the ACF had higher cost-effectiveness, semi-PCF has the strength of a low WTP. As shown in Fig 2, indirect costs of ACF and semi-PCF have a higher impact than total cost of treatment because a significantly smaller proportion of total participants would undergo treatment. Comparing ACF and semi-PCF, costs for mobile screening equipment are included in ACF performed by NGOs; therefore, this indirect cost could have been underestimated, even though the real cost could be much increased under the actual routine situation [27].

This study has several limitations. Firstly, the reference of our study is based on immigrants who reside in densely-populated areas and might not be representative. Secondly, in our research, we assumed that 99% of SC-proven active TB patients underwent treatment, and every strain of active TB was drug-susceptible because of the ignorance about the flow and outcome of multidrug-resistant tuberculosis among immigrants [3]. Therefore, the definite treatment outcome of TB including MDR-TB for immigrants should be input into a future cost-effectiveness study. Last, while we calculated the TB treatment cost, we assumed that the treatment was performed only in an outpatient clinic, and micro-costing for calculating indirect costs was not used.

Nevertheless, considering the importance of communicable disease control against respiratory infections, the significance of our study is the cost-effective analysis based on actual data about the difference between ACF and semi-PCF in TB among vulnerable immigrants. In order to establish an appropriate TB screening policy for immigrants, in-depth consideration and investigation of the economic circumstances of public healthcare as well as of the social characteristics of the screening target group are warranted.

## Conclusion

As TB screening policies, ACF and semi-PCF show several differences, including the target group’s average age, region of origin, visa type, and CXR-positive ratio. On cost-effectiveness analysis based on real data and literature review, ACF can be preferred based on cost-effectiveness. However, in-depth investigation of costs and the target group will be needed in prospective studies to establish a more cost-effective immigrant TB screening strategy.

## Supporting information

S1 Fig Probabilistic sensitivity analysis with 1,000 Monte Carlo simulations at a WTP threshold of $1,500. WTP = willingness-to-pay.
(TIF)
