# Supplementary figures and images for: Comparison of active tuberculosis case finding strategies for immigrants in South Korea: Epidemiology and cost-effectiveness analysis
Source: PLoS One. 2023 Apr 20;18(4):e0283414. doi: 10.1371/journal.pone.0283414 (PMC10118078; doi:10.1371/journal.pone.0283414)

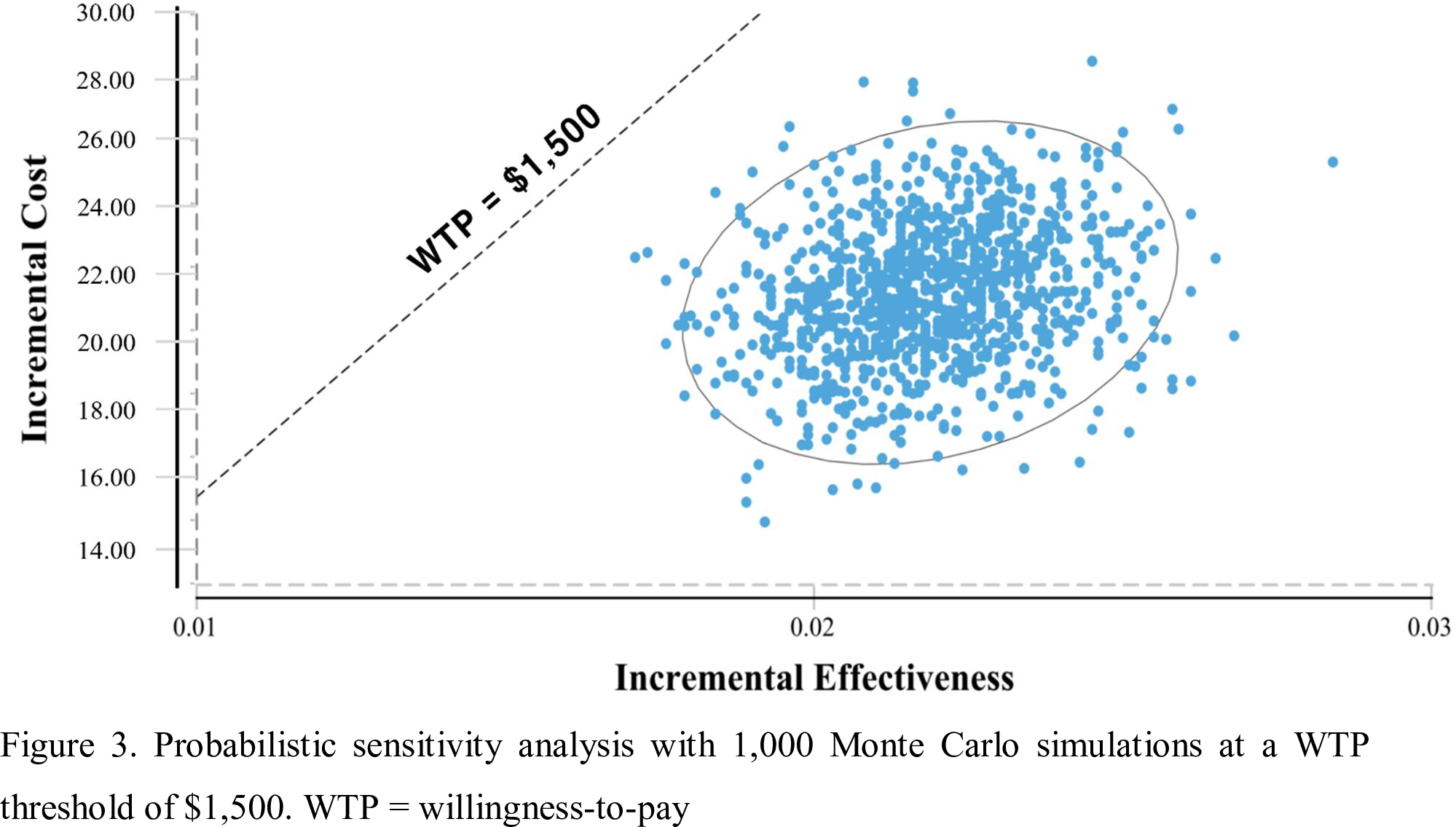

Supplement: S1 Fig — WTP = willingness-to-pay. (TIF) [file pone.0283414.s001.tif]
